# An *in vitro* comparative study on the flexural strength of different restorative materials

**DOI:** 10.6026/973206300221901

**Published:** 2026-03-31

**Authors:** Mahboob Rahaman S.K, Pranjali Dutt, Kanchan Sharma, Shreya Gill, Jay Gohil, Vaishali Ulhas Bhalerao, Pratik Surana

**Affiliations:** 1 Department of Conservative Dentistry & Endodontics, North Bengal Dental College & Hospital, Darjeeling, West Bengal, India; 2 Department of Dentistry, Mahamaya Rajkiya Allopathic Medical College, Ambedkarnagar, Uttar Pradesh, India; 3 Department of Orthodontics and Dentofacial Orthopaedics, Awadh Dental College and Hospital, Jamshedpur, Jharkhand, India; 4 Department of Conservative Dentistry and Endodontics, Winchester Dental Care, Virginia, USA; 5 Department of Prosthodontics and Crown & Bridge, K.M Shah Dental College and Hospital, Sumandeep Vidyapeeth deemed to be University, Waghodia, Vadodara, Gujarat, India; 6 Department of Conservative Dentistry and Endodontics, Rural Dental College, Pravara Institute of Medical Sciences (DU), Loni Bk. Tal-Rahata, Ahmednagar, India; 7 Department of Pedodontics and Preventive Dentistry, Maitri College of Dentistry and Research Centre, Durg, Chhattisgarh, India

**Keywords:** Glass ionomer cement, Amalgomer CR, flexural strength

## Abstract

Failure of restorative materials under masticatory stress remains a persistent problem in restorative dentistry, particularly in load- bearing areas. Flexural strength is an important mechanical property that influences the fracture resistance and durability of restorative materials. Therefore, it is of interest to evaluate the flexural strength of conventional glass ionomer cement, Type IX glass ionomer cement, and ceramic-reinforced glass ionomer cement (Amalgomer CR). Standardized specimens tested with a universal testing machine showed a significant difference among groups (p < 0.001), with ceramic-reinforced GIC exhibiting the highest flexural strength.

## Background:

Glass ionomer cement (GIC) was first formulated by Wilson and Kent in the early 1970s as a hybrid restorative material that integrates the advantageous properties of both silicate and polycarboxylate cements [1]. Since its introduction, GIC has gained wide acceptance in dentistry due to its chemical adhesion to enamel and dentin, fluoride-releasing ability, biocompatibility, and thermal expansion coefficient comparable to that of natural tooth structure [2]. These characteristics make GIC particularly useful in atraumatic restorative treatment, pediatric dentistry, and preventive restorative procedures [3]. However, despite these advantages, conventional GICs exhibit limitations related to their mechanical properties, especially low tensile and flexural strength, which restrict their use in stress-bearing areas [4]. Flexural strength is an essential mechanical property that indicates a restorative material's capacity to withstand deformation and fracture when subjected to bending forces during mastication [5]. Clinically, restorations are subjected to complex stresses rather than simple compressive loads; therefore, flexural strength is considered a more relevant indicator of long-term clinical performance and durability [6]. Low flexural strength may result in marginal breakdown, crack propagation, and restoration failure, emphasizing the need for improved formulations of GIC with enhanced mechanical properties [7]. To address the limitations of conventional glass ionomer cements (GICs), high-viscosity GIC such as Type IX GIC were developed. Type IX GIC is characterized by a higher powder-to-liquid ratio, improved handling characteristics, faster setting time, and enhanced mechanical strength compared to conventional GICs. These properties have expanded its clinical applications to include posterior restorations in low to moderate stress-bearing areas [8]. Further advancements led to the introduction of ceramic-reinforced glass ionomer cements, such as Amalgomer CR, which incorporate ceramic fillers into the glass matrix. The addition of ceramic particles aims to improve flexural strength, fracture resistance, and wear properties while retaining the fundamental benefits of GICs, including fluoride release and adhesion. Ceramic-reinforced GICs have been proposed as alternatives to amalgam in posterior restorations, particularly where improved strength is required [9, 10]. Therefore, it is of interest to evaluate the flexural strength of conventional, Type IX, and ceramic-reinforced GIC to determine their suitability for different clinical applications.

## Materials and Methods:

The objective of this *in vitro* study was to analyze the flexural strength of three varieties of GIC: conventional GIC (Group I: GC Corporation, Japan), Type IX GIC (Group II, GC Corporation, Japan), and ceramic-reinforced glass ionomer cement (Group III; Amalgomer CR, Advanced Healthcare Ltd. United Kingdom).

## Study design and sample size:

A total of 30 test units were prepared and equally allocated into three groups (n = 10). The sample size was determined from previous *in vitro* studies evaluating flexural strength of glass ionomer cements and was considered adequate for statistical comparison.

## Specimen preparation:

Flexural strength test samples fabricated using a standardized stainless steel mold measuring 25 mm x 2 mm x 2 mm, in accordance with ISO 4049 specifications for flexural strength testing. The mold was positioned on a glass slab and filled with the freshly mixed material. Mixing was carried out manually on a mixing pad using a plastic spatula, following the powder-to-liquid ratio recommended by the manufacturer for each material. After filling, a second glass slab was placed over the mold, and gentle pressure was applied to remove excess material and achieve a smooth surface. The specimens were allowed to set at room temperature and were subsequently removed from the mold after completion of the initial setting.

## Storage Conditions:

All samples were stored in distilled water at 37°C for 24 hours, then subjected to thermocycling (500 cycles between 5°C and 55°C with a dwell time of 30 seconds) to simulate oral conditions prior to testing.

## Flexural strength testing:

Flexural strength was assessed using a universal testing machine by subjecting each specimen to a three-point bending test. A load was applied at the midpoint of the specimen at a crosshead speed of 1 mm/min until fracture occurred, and the maximum fracture load was recorded in Newtons (N),

Flexural strength (MPa) was calculated using:

Flexural strength = 3FL / 2bd^2^,

Where,

F = fracture load (N)

L = support span (mm)

b = specimen width (mm)

d = thickness (mm).

## Statistical analysis:

Data were recorded and analyzed using statistical software. Mean flexural strength were calculated for each group, with intergroup comparisons made using one-way ANOVA and post-hoc Tukey's test. A p-value < 0.05 was considered significant.

## Results:

The mean flexural strength values of the three glass ionomer cements evaluated in this study are presented in Table 1 (see PDF). Group I (conventional GIC) exhibited the lowest flexural strength (18.6 ± 2.4 MPa), followed by Group II (Type IX GIC) with a moderate increase (27.9 ± 3.1 MPa), while Group III (ceramic-reinforced GIC, Amalgomer CR) demonstrated the highest flexural strength (38.5 ± 3.8 MPa). Intergroup comparison revealed statistically significant differences in flexural strength among all three groups (Table 2 - see PDF), with Amalgomer CR showing significantly higher values than both conventional and Type IX GIC, and Type IX GIC demonstrating superior strength compared to conventional GIC. Flexural strength is a critical structural characteristic that reflects the ability of restorative materials to withstand tensile stresses generated during mastication and functional loading [11]. Since intraoral forces are predominantly complex and multidirectional rather than purely compressive, flexural strength is considered a reliable indicator of the clinical durability of glass ionomer cement restorations [12]. The findings of the present *in vitro* study clearly demonstrate statistically significant differences in flexural strength among conventional GIC, Type IX GIC, and ceramic-reinforced GIC. The lowest mean flexural strength observed with conventional GIC is in agreement with previous reports that describe its inherent brittleness and limited resistance to tensile and bending stresses. This behavior has been attributed to its porous structure and weak matrix-filler bonding [13], which restricts its application in stress-bearing areas despite advantages such as fluoride release and chemical adhesion to tooth structure. Type IX GIC showed significantly higher flexural strength than conventional GIC. This improvement may be explained by its high-viscosity formulation, increased powder-to-liquid ratio, and enhanced glass particle content, which collectively improve mechanical interlocking and reduce porosity [14]. Similar observations were reported by Xie *et al.* (2000), who found that high-viscosity GICs exhibit superior mechanical properties, making them suitable for posterior restorations in low- to moderate stress-bearing regions [16]. The highest flexural strength was recorded for Amalgomer CR, which demonstrated statistically significant superiority over both conventional and Type IX GICs. The incorporation of ceramic particles into the glass matrix enhances crack resistance, load distribution, and fracture toughness. Ceramic fillers function as stress-bearing components that impede crack propagation, thereby markedly improving flexural strength [16]. These findings are consistent with those of Chowdhary *et al.* (2019) and Lohbauer *et al.* (2006), who reported that ceramic reinforcement significantly enhances the mechanical performance of GICs without adversely affecting their adhesive properties or fluoride release [17, 18]. Overall, the progressive increase in flexural strength from conventional GIC to Type IX GIC and further to ceramic-reinforced GIC underscores the importance of material modification in improving the mechanical behavior of glass ionomer cements. From a clinical perspective, ceramic-reinforced GICs may serve as promising alternatives to traditional restorative materials in posterior restorations requiring higher strength, particularly in patients with moderate occlusal loads. Nevertheless, the present study has certain limitations. As an *in vitro* investigation, it does not fully simulate the oral environment, where factors such as thermal cycling, moisture variations, pH changes, and long-term fatigue loading may influence material performance. Therefore, further studies incorporating aging protocols and well-designed clinical trials are recommended to validate these findings and assess long-term clinical outcomes. Restorative materials are expected to closely mimic the natural tooth in both esthetic and physical properties. Their ability to withstand masticatory forces, particularly compressive and flexural stresses, plays an important role in determining the longevity and clinical success of restorations [19, 20]. Flexural strength reflects the material's ability to resist deformation and fracture under bending forces during mastication [21, 22]. However, other variables such as wear resistance, bonding ability, polymerization shrinkage, and marginal integrity also influence the overall clinical performance of restorative materials [23]. Further studies are recommended to evaluate these variables to obtain a more comprehensive understanding of restorative material performance.

## Conclusion:

We show ceramic-reinforced glass ionomer cement showed superior flexural strength over conventional and Type IX glass ionomer cements. Thus, we show modified GICs may be more suitable for clinical situations requiring enhanced mechanical durability.

## Advancement to knowledge:

This study provides comparative data on the flexural strength of different restorative materials (conventional glass ionomer cement, Type IX glass ionomer cement, and ceramic-reinforced glass ionomer cement), highlighting differences in their mechanical performance. The findings may assist clinicians in selecting materials with greater resistance to fracture in stress-bearing restorations.
